# The Mechanism and Function of miRNA in Intervertebral Disc Degeneration

**DOI:** 10.1111/os.13204

**Published:** 2022-02-09

**Authors:** Chenglong Wang, Liqiang Cui, Qinwen Gu, Sheng Guo, Bin Zhu, Xueli Liu, Yujie Li, Xinyue Liu, Dingxuan Wang, Sen Li

**Affiliations:** ^1^ Spinal Surgery Department The Affiliated Traditional Chinese Medicine Hospital of Southwest Medical University Luzhou China; ^2^ Department of Spine Surgery Mianyang Orthopaedic Hospital Mianyang China; ^3^ Institute of Physical Education Southwest Medical University Luzhou China

**Keywords:** Apoptosis, Inflammatory factors, Intervertebral disc degeneration, miRNA, Proliferation

## Abstract

Intervertebral disc degeneration (IDD) disease has been considered as the main cause of low back pain (LBP), which is a very common symptom and the leading cause of disability worldwide today. The pathological mechanism of IDD remains quite complicated, and genetic, developmental, biochemical, and biomechanical factors all contribute to the development of the disease. There exists no effective, non‐surgical treatment for IDD nowadays, which is largely related to the lack of knowledge of the specific mechanisms of IDD, and the lack of effective specific targets. Recently, non‐coding RNA, including miRNA, has been recognized as an important regulator of gene expression. Current studies on the effects of miRNA in IDD have confirmed that a variety of miRNAs play a crucial role in the process of IDD via nucleus pulposus cells (NPC) apoptosis, abnormal proliferation, inflammatory factors, the extracellular matrix (ECM) degradation, and annulus fibrosus (AF) degeneration. In the past 10 years, research on miRNA has been quite active in IDD. This review summarizes the current research progression of miRNA in the IDD and puts forward some prospects and challenges on non‐surgical treatment for IDD.

## Graphical abstract

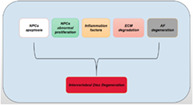

A variety of miRNAs have been confirmed that they play a crucial role in the process of intervertebral disc degeneration (IDD) via nucleus pulposus cells apoptosis, abnormal proliferation, inflammatory factors, the extracellular matrix degradation, and annulus fibrosus degeneration. We summarized the mechanism and function of miRNA in IDD and put forward some prospects and challenges on miRNA‐related treatment for IDD.

## Introduction

**Fig. 1:**
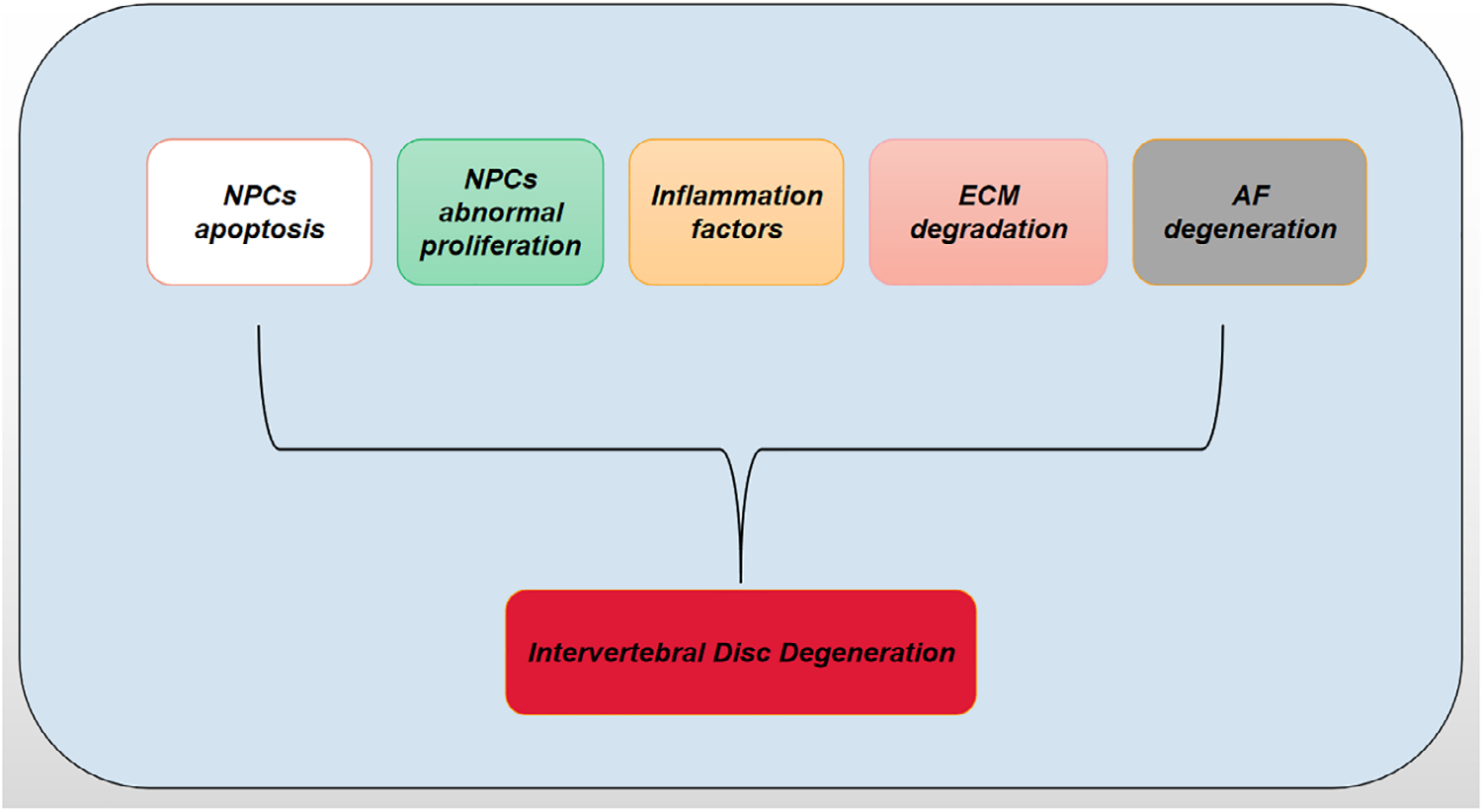
NPCs apoptosis, abnormal proliferation, inflammatory factors, ECM degradation, and annulus fibrosus degeneration all lead to and accelerate the process of IDD.

Intervertebral disc degeneration (IDD) has been considered the main cause of low back pain (LBP) and places a heavy burden on the global healthcare system^1^. The intervertebral disc is composed of the nucleus pulposus (NP) and the annulus fibrosus (AF), which can bear and relieve the pressure on the spine together^2^. As the main cause of LBP, the pathogenesis of IDD remains quite complicated. There are many factors that can promote or accelerate IDD, such as genes, age, and bad living habits including occupational factors, smoking, and alcoholism^3^. Moreover, there is no good non‐surgical treatment strategy to reverse IDD now, which is largely due to the unclear specific mechanism of IDD and the lack of effective specific targets. In recent years, numerous studies found that degeneration‐related genes play an important role in the process of IDD, but the mechanism is still unclear. As one of the important regulatory molecules of gene expression, miRNA has been shown to play an absolutely key role in the initiation and progression of various diseases. Published articles have confirmed that miRNA is involved in the process of IDD^4^. Current research has found that a variety of miRNAs participate in the process of IDD via NPC apoptosis, NPC abnormal proliferation, inflammatory factors, ECM degradation, and AF degeneration^5^ (Figure 1).

### Search Strategy

**Fig. 2:**
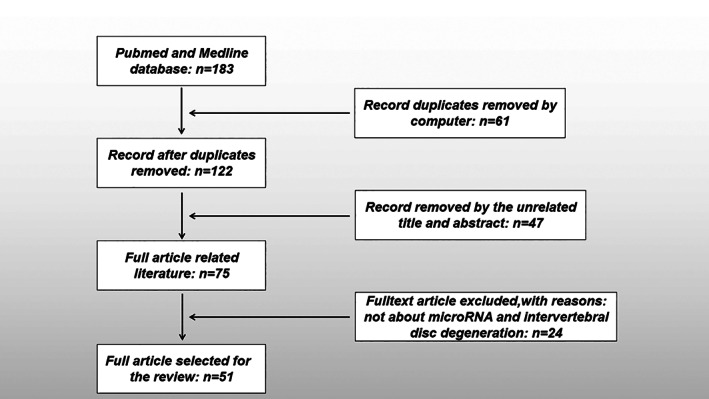
Flow chart of the seach for published articles showing the process of inclusion and exclusion.

(i) **Searching platforms:** Articles were hand‐searched from PubMed, a retrieval biomedical literature database.
(ii) **Databases:** MEDLINE, OLDMEDLINE.
(iii) **Keywords:** microRNA; miRNA; intervertebral disc degeneration; IDD; degenerated intervertebral disc.
(iv) **Boolean algorithm:** microRNA AND intervertebral disc degeneration.
(v)   **Retrieving time:** Issues of the selected journals published from 2011 to 2021 were hand‐searched by us.
(vi) **Inclusion and exclusion criteria:** The inclusion criteria for articles are: (i) studies related to microRNA and intervertebral disc degeneration; (ii) article types are monographs, research papers, and reviews. The exclusion criteria are repetitive research and unavailable full text. The search process was performed as presented in Figure 2.

## Intervertebral Disc and Intervertebral Disc Degeneration

The intervertebral disc is located between the vertebral bodies of the spine. It is cylindrical and consists of the upper and lower cartilage endplates, the annulus fibrous, and the surrounding jelly‐like nucleus pulposus. The intervertebral disc has the biomechanical functions of maintaining the stability of the spine, absorbing and buffering shocks, and equalizing external forces. In the process of IDD, the intervertebral disc undergoes complex biochemical and molecular changes. These changes include the reduction of proteoglycan content, the conversion of type II collagen (COL II) to type I collagen (COL I), and the decrease of NPC density. They can directly lead to the decrease in the mechanical action of the intervertebral disc and destruction of the structure, such as annulus fibrous rupture, nucleus pulposus herniation, etc. In addition, the release of inflammatory factors, extracellular matrix decomposition, and synthesis also participate in the degenerative process^6^. However, the specific molecular mechanism and function are still unclear, which leads to many limitations on the non‐surgical treatment research of IDD.

## miRNA

As a short non‐coding RNA, microRNA (miRNA), was officially recognized as one of the classic gene regulators in eukaryotic cells in 2001^7^. And as an endogenous small RNA, miRNA plays an important role in cell proliferation, development, and metabolism by acting on other genes^8^. MiRNA is a non‐coding region, single‐stranded RNA composed of 18–22 nucleotides, which is formed by pri‐miRNA transcription. It is generally believed that pri‐miRNA has two sources: (i) genes encoded by special miRNAs are transcribed through II Type RNA polymerase, and then these pri‐miRNAs are cleaved in the nucleus through the interaction of multiple proteins. These proteins contain an ankyrin DGCR8, which can contribute to pre‐miRNA composed of approximately 70 nucleotides; (ii) transcribed from the internal fragments of mRNA, their maturation process does not require the participation of Drosha/DGCR8. These miRNAs are separated and spliced by Lariat Debranching Enzyme and share with the host protein‐coding gene transcription to form hairpin‐like pre‐miRNAs; these internal miRNAs often appear in the same biological pathway as the gene encoding the host protein. Researchers^9^ have found that many abnormally expressed miRNAs are expressed in degenerative intervertebral disc tissues, suggesting that miRNAs may be involved in the pathophysiological process of IDD.

## MiRNA in IDD

### NPCs Apoptosis

**Fig. 3:**
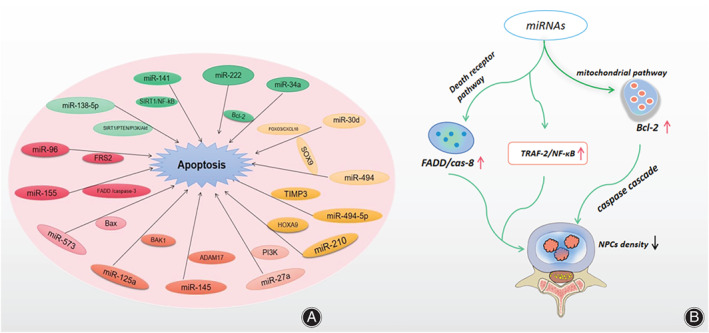
(A) MiRNA participates in IDD by promoting apoptosis of NPCs. (B) By activating the mitochondrial pathway, miRNA increases mitochondrial membrane permeability, loss of transmembrane potential, release of cytochrome C and other proteins, which ultimately increases apoptosis and decreases density of NPCs via a series of caspase cascades. In addition, miRNA activates death receptor pathways, such as FADD/cas‐8 and TRAF‐2/NF‐kB, which can also increase apoptosis of NPCs. Red arrow indicates the rising level or amount while blue arrow in contrast and same below. Abbreviations: Bax (bcl2‐associated x), MMP (mitochondrial membrane permeability), TRAF‐2 (TNF receptor associated factor 2), NF‐kB (Nuclear factor‐kappa B).

Normal apoptosis can maintain the stability of the internal environment, and the process is the autonomous programmed cell death controlled by genes. Both exogenous and endogenous pathways play an important role in the process of human NPCs apoptosis. The excessive apoptosis of NPCs reduces the density of NPCs in the intervertebral disc tissue, destroys the structure and function of the intervertebral disc, and leads to degeneration of intervertebral disc. The major exogenous signaling pathway is the FasL—Fas signaling pathway, which is composed of the Fas‐related death domain‐containing protein (FADD) and caspase‐3 pathway^10^. The endogenous apoptosis signaling pathway originates from mitochondria and involves the classic anti‐apoptotic protein B‐cell lymphoma leukemia‐2 (Bcl‐2) family^11^ (Figure 3A).

Wang *et al*.^12^ found that miR‐138‐5p was significantly up‐regulated in IDD tissues, and the inhibited miR‐138‐5p can reduce the apoptosis induced by TNF‐α. Knockout of miR‐138‐5p can protect human NPCs from excessive apoptosis caused by SIRT1 upregulation, which is mediated by PTEN/PI3K/Akt signaling pathway. High expression of miR‐143‐5p^13^ or activation of AMPK signaling pathway inhibited the proliferation and differentiation of NPCs and promoted the apoptosis and senescence of NPCs. Ji *et al*.^14^ confirmed that miR‐141 drove IDD by inducing apoptosis of NPCs, which directly targeted to SIRT1/NF‐kB pathway to partly promote the process of IDD. In vitro, knocking out miR‐141 can attenuate IDD induced spontaneously or surgically. And they delivered the up or down‐regulated miR‐141 through nanoparticles in the IDD rat model to aggravate or reduce experimental IDD. MiR‐222^15^ was significantly up‐regulated in degenerative NPCs. And the overexpressed miR‐222 can activate Bax and caspase 3 but inhibit Bcl‐2. It is worth noting that the activation of Bax and caspase 3 can promote apoptosis, while the activation of Bcl‐2 can inhibit apoptosis. Chen *et al*.^16^ found that miR‐34a was significantly increased apoptosis in human degenerated CEP chondrocytes by targeting Bcl‐2. And knockdown of miR‐34a led to overexpression of Bcl‐2, which can reduce apoptosis, while upregulation of miR‐34a had an opposite effect in human CEP cells. MiR‐30d^17^ was highly expressed in human IDD tissues. And down‐regulated miR‐30d promoted the proliferation of degenerated NPCs and inhibited apoptosis by targeting the FOXO3/CXCL10 axis.

Moreover, miR‐96 promoted apoptosis of NPCs by targeting FRS2, miR‐494 promoted apoptosis of NPCs by targeting SOX9, miR‐494‐5p enhanced the viability of NPCs and reduced apoptosis and senescence by increasing TIMP3 in mice, miR‐30d can promote NPCs viability and reduce apoptosis by targeting SOX9, miR‐210 may promote Fas‐mediated apoptosis of NPCs by regulating the expression of HOXA9, miR‐27a can accelerate NPCs apoptosis by targeting PI3K, miR‐145 attenuated NPCs apoptosis by targeting ADAM17, miR‐125a may regulate the apoptotic state of NPCs by inhibiting BAK1, miR‐573 can inhibit NPCs apoptosis by inhibiting Bax and miR‐155 promoted Fas‐mediated apoptosis of NPCs by targeting FADD and caspase‐3^18^, ^19^, ^20^, ^21^, ^22^, ^23^, ^24^, ^25^, ^26^, ^27^_._

### Abnormal NPCs Proliferation

**Fig. 4:**
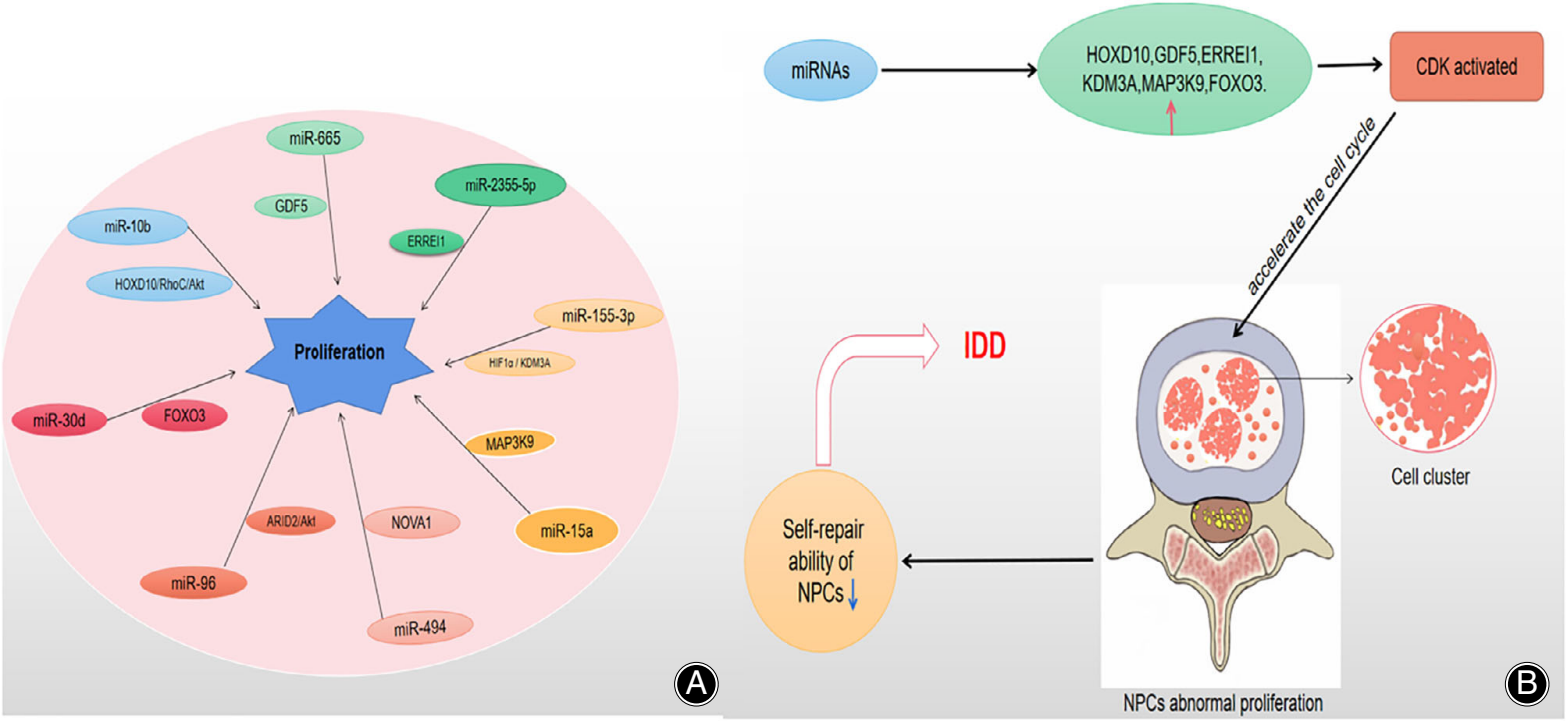
(A) MiRNAs participate in IDD via NPCs abnormal proliferation. (B) MiRNAs activate CDK, which can accelerate the cell cycle and cause the abnormal proliferation of NPCs. Abnormal proliferation inhibits the self‐repair ability of NPCs by forming cell clusters. Abbreviation: CDK (cyclin dependency kinase).

The abnormal proliferation of NPCs is closely related to the progress of IDD. One of the pathological features of IDD is the abnormal proliferation of NPCs, which inhibit the self‐repair ability of intervertebral disc cells by forming cell clusters^28^ (Figure 4A,B).

MiR‐10b^29^ was significantly up‐regulated in IDD tissue, which is related to the down‐regulation of HOXD10. In vitro, the overexpressed miR‐10b stimulated the proliferation of NPCs and translational inhibition of HOXD10, while the restored expression of HOXD10 reversed the mitogenic effect of miR‐10b. In short, down‐regulated HOXD10 can stimulate RhoC expression and phosphorylation. And knockout of RhoC or inhibition of Akt can eliminate the effect of miR‐10b on the proliferation of NPCs. Tan *et al*.^30^ found that the expression level of miR‐665 was positively correlated with Pfirrmann grade and the highly expressed miR‐665 promoted the abnormal proliferation of NPCs by targeting growth differentiation factor 5 (GDF5) in IDD tissues. As a target of miR‐2355‐5p^31^, ERREI1 (product of mitogen‐inducible gene 6) was low‐expressed in IDD tissue and NPCs were in a state of excessive proliferation when miR‐2355‐5p was high‐expressed. In contrast, ERREI1 inhibited the excessive proliferation of NPCs and delayed the process of IDD when miR‐2355‐5p was down‐regulated. In IDD tissues, up‐regulated miR‐155‐3p or silent KDM3A can promote the proliferation of NPCs by inhibiting HIF1α^32^. MiR‐15a^33^ was significantly up‐regulated in IDD tissues and overexpressed miR‐15a can promote abnormal proliferation of NPCs by inhibiting MAP3K9.

In addition, miR‐494^34^ regulated the proliferation of NPCs by targeting NOVA1, miR‐96^35^ promoted the abnormal proliferation of NPCs by activating the ARID2/Akt pathway, the down‐regulated miR‐30d^17^ promoted the proliferation of NPCs by activating FOXO3 and inhibiting CXCL10.

### Inflammatory Factors

**Fig. 5:**
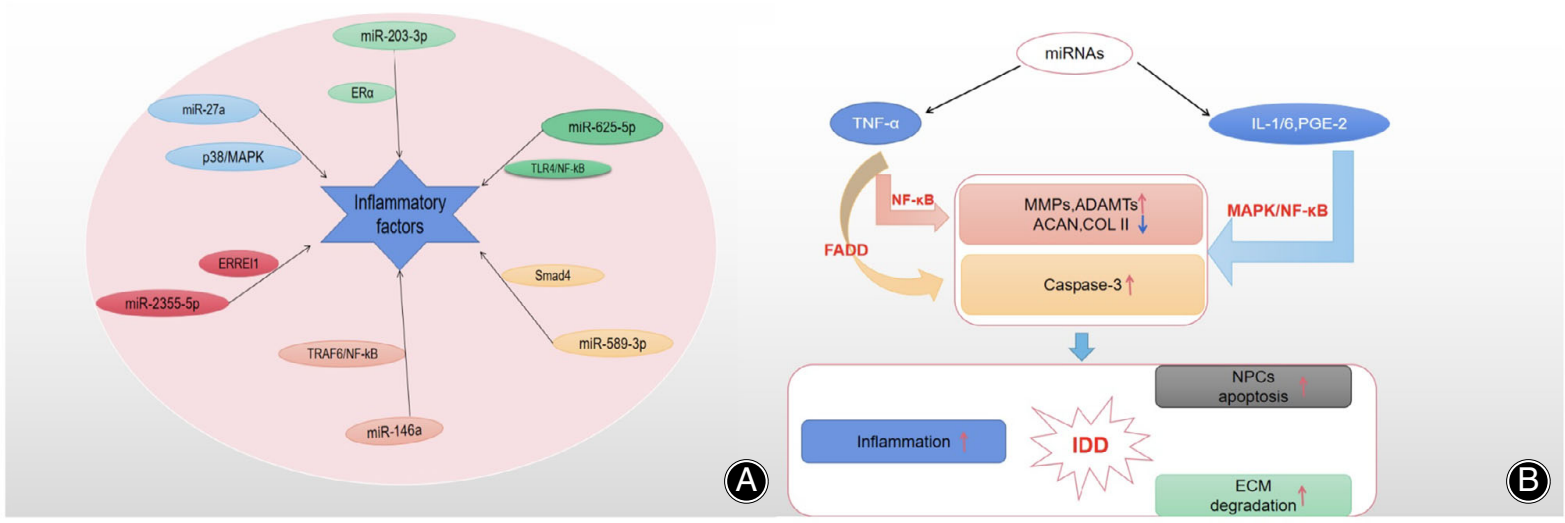
(A) MiRNAs participate in IDD through inflammation signaling pathways. (B) MiRNAs activate inflammation‐related pathways, such as TNF, IL, PGE2, which can promote the apoptosis of NPCs, accelerate the degradation of ECM and maintain NPCs in an inflammation cascade. Abbreviations: TNF (Tumor necrosis factor), IL (Interleukin), PEG2 (Prostaglandin E2).

A large number of studies have shown that inflammation factors play a key role in the process of IDD. The interaction and abnormal expression of inflammatory factors can disrupt the balance of extracellular matrix metabolism, cause inflammation, and accelerate IDD^36^. Tumor necrosis factor (TNF), interleukin (IL), nitric oxide, and prostaglandin E2 (PGE2) are the main factors for the inflammatory reaction in intervertebral disc tissue^37^. Current research suggests that miRNA can speed up or delay the process of IDD via inflammatory factors (Figure 5A,B).

MiR‐27a^38^ was significantly up‐regulated in the inflammatory IDD model established by LPS stimulation. When miR‐27a expression was inhibited, the expression of both p‐p38/NF‐kB and the pro‐inflammatory factors IL‐1β, IL‐6, and TNF‐α were inhibited. And low‐expressed miR‐27a can inhibit the release of pro‐inflammatory factors through the p38/MAPK signaling axis. In high‐level degenerative NP tissue, miR‐203‐3p^39^ was significantly up‐regulated and negatively correlated with the expression of estrogen receptor α (ERα). In the inflammatory mice model, the expression of miR‐203‐3p was up‐regulated but ERα down‐regulated. Low‐expressed miR‐203‐3p can inhibit inflammation response and degeneration by targeting ERα. Activation of the TLR4/NF‐kB signaling pathway increased the level of pro‐inflammatory factors and the expression of miR‐625‐5p but decreased the expression of COL1A1^40^. Up‐regulated miR‐589‐3p^41^ increased the apoptosis rate of NPCs and the production of pro‐inflammatory factors TNF‐α, IL‐1, and IL‐6, reduced the expression of COL II and aggrecan (ACAN) by inhibiting Smad4. In the peripheral blood mononuclear cells (PBMC) of IDD patients, miR‐146a^42^ was in a low expression level. While in the IDD inflammatory rat model, the up‐regulated miR‐146a suppressed the mRNA and protein levels of TRAF6/NF‐kB, and significantly reduced the levels of pro‐inflammatory cytokines in NPCs. In addition, miR‐2355‐5p^31^ can inhibit the production of pro‐inflammatory factors through regulating ERREI1 negatively.

### ECM Degradation

**Fig. 6:**
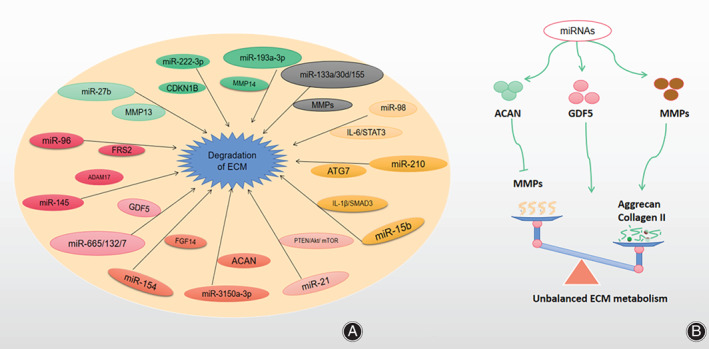
(A) MiRNAs participate in IDD via promoting ECM degradation or synthesis. (B) MiRNA increases the expression of MMPs and ADAMTSs and decreases the expression of proteoglycans and collagen via activating ECM‐related signaling pathways, which remains more degradation than synthesis. Inhibiting the activation of ECM‐related signaling pathways can keep degradation and synthesis in a balanced state, which can delay or even reverse the IDD process. Abbreviations: MMP (Matrix metalloproteinase), ADAMTS (A disintegrin and metalloproteinase with thrombospondin‐like motifs).

ECM is the microenvironment where cells produce and live, which is composed of collagen, proteoglycans, non‐collagen, elastic fibers, water, and glycoproteins. And the major components are glycoproteins and COL II, which combine with water to provide expansion force to resist the compression of the intervertebral disc and prevent excessive water loss^43^. Numerous published articles have confirmed that the main pathological characteristic of IDD is the loss of collagen and proteoglycan in the intervertebral disc. Matrix metalloproteinases (MMPs) are a family of zinc‐containing proteolytic enzymes that are widely present in the human body. They can degrade most of the extracellular matrix components. Till now, 28 kinds of matrix metalloproteinases have been discovered. MMPs and a disintegrin and metalloproteinase with thrombospondin motifs (ADAMTSs) are the main enzymes that degrade collagen and proteoglycans. Their combined action reduces the extracellular matrix content and leads to degeneration of the intervertebral disc^44^ (Figure 6A,B).

As the target of miR‐27b, MMP13 was negatively regulated by miR‐27b. And the down‐regulated miR‐27b accelerated the loss of COL II by directly targeting MMP13^45^. The up‐regulated miR‐222‐3p^46^ promoted the secretion of MMP3 and reduced the content of COL II and ACAN by directly regulating cyclin‐dependent kinase 1B (CDKN1B) negatively. Research^47^ has confirmed that the down‐regulated miR‐193a‐3p can promote the process of IDD via increasing the expression of MMP14 and accelerating the loss of COL II in vivo and in vitro. MiR‐133a^48^ was the most significantly down‐regulated in 31 miRNAs differentially expressed in IDD tissues and the down‐regulated miR‐133a induced the loss of COL II in NPCs through directly targeting MMP9. Ji *et al*. found that miR‐98^49^ remained significantly down‐regulated in IDD tissues and IL‐6 was confirmed to be a target of miR‐98. The level of IL‐6 mRNA was negatively correlated with miR‐98 and the IL‐6 treatment can eliminate the high‐expression of COL II stimulated by up‐regulated miR‐98. In addition, miR‐98 can significantly inhibit the expression of MMP2. They believed that the down‐regulation of miR‐98 may promote IDD through the IL‐6/STAT3 signaling pathway. MiR‐210^50^ can inhibit autophagy by silencing autophagy‐related gene 7 (ATG7), which leads to the increasing expression of MMP‐3 and MMP‐13, decreasing expression of COL II and ACAN in degenerated NPCs. Overexpressed miR‐15b^51^ aggravated the ECM degradation of NPCs induced by IL‐1β. Inhibiting miR‐15b can delay the degenerated process by targeting SMAD3, which is the key mediator of the conduction pathway of transforming growth factor‐β. MiR‐21^52^ can inhibit autophagy, up‐regulate the expression of MMP‐3 and MMP‐9, increase degradation of COL II and ACAN through the PTEN/Akt/mTOR signal transduction pathway. Zhang *et al*.^53^ found that the up‐regulated miR‐3150a‐3p can reduce the expression of ACAN in degenerative NPCs, while the down‐regulated miR‐3150a‐3p can reverse the low expression of ACAN.

In addition, miR‐154^54^ promoted ECM degradation in IDD by targeting fibroblast growth factor 14 (FGF14). miR‐665,^30^ miR‐132^55^, and miR‐7^56^ all accelerated human NPCs ECM degradation by targeting GDF5. The overexpressed miR‐145^24^ increased ECM synthesis by targeting ADAM17. The up‐regulated miR‐30d^21^ reduced the expression of COL II and proteoglycan via targeting MMP. The down‐regulated miR‐155^57^ degraded proteoglycans and COL II and led to degeneration of intervertebral discs by targeting MMP‐16.

### AF Degeneration

**Fig. 7:**
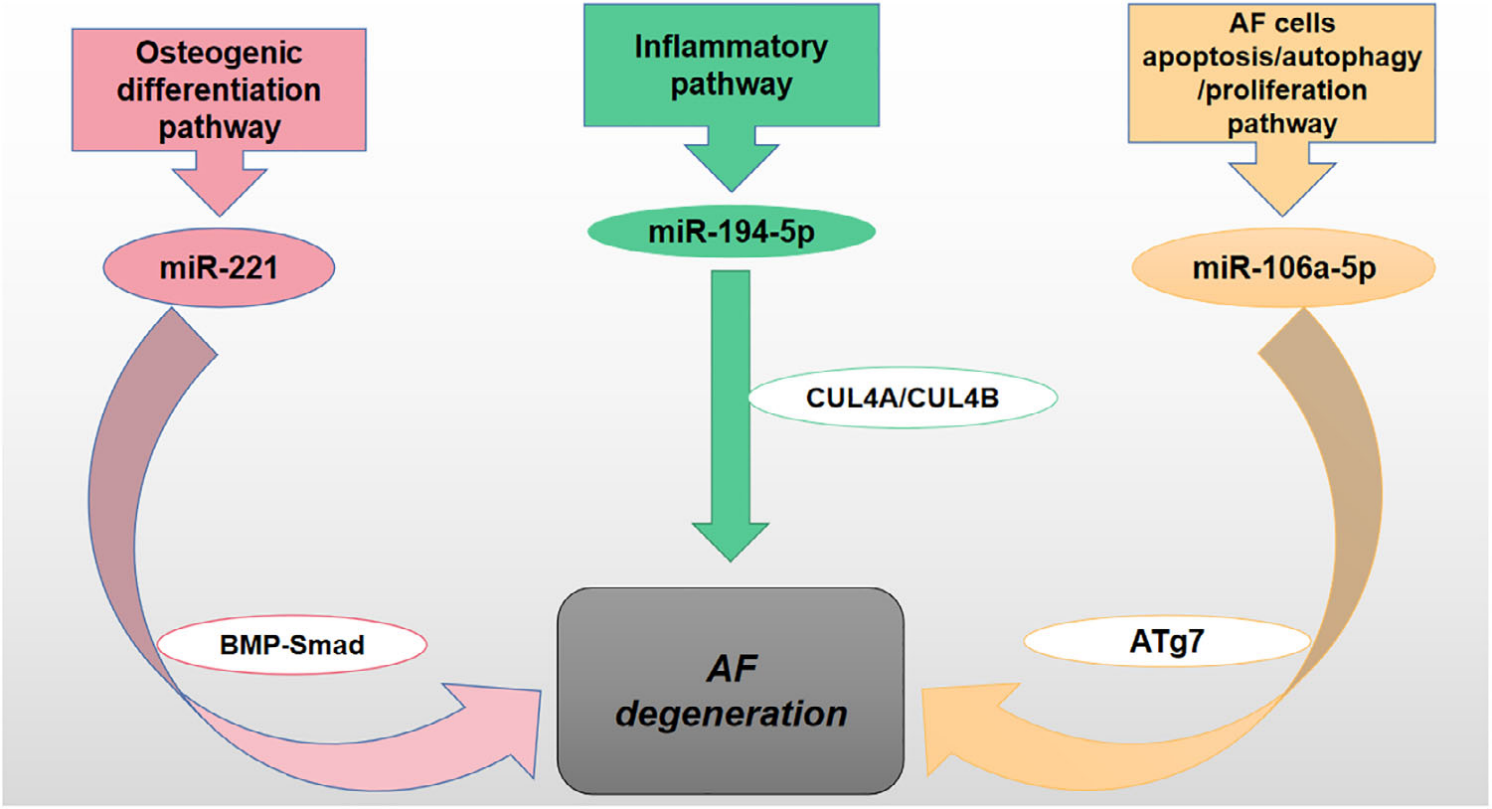
MiRNA participates in the degeneration of AF through the following three pathways: miRNA regulates the BMP‐Smad pathway to promote the osteogenic differentiation of AF cells; MiRNA participates in AF degeneration through inflammatory signaling pathways regulated by CUL4A and CUL4B; MiRNA participates in the abnormal proliferation, apoptosis, and autophagy via regulating ATg7. Abbreviations: BMP (Bone morphogenetic protein), CUL4A (Cullin4A), ATg7 (Autophagy‐related genes 7).

The annulus fibrosus is composed of fibrocartilage and is located on the periphery of the intervertebral disc. When the nucleus pulposus is slightly flattened under pressure, the tightly arranged annulus fibers can absorb the pressure from the nucleus pulposus to the AF wall. Therefore, the degeneration of AF often develops from the degeneration of NP. Within the limit of physiological stress, the annulus fibrosis can disperse and slow down the stress of the nucleus pulposus‐vertebral body‐spine. However, when the pressure is greater than the limit of physiological stress, it may cause damage to the annulus, such as annulus fibrous rupture. Similar to the degeneration of NP, miRNA has also been confirmed to be involved in the degeneration process of AF (Figure 7).

Yeh *et al*.^58^ found that the basal level of miR‐221 in degenerated AF cells was significantly reduced and the degenerated AF cells had a greater tendency to osteogenic differentiation through activating BMP‐Smad pathway. Smads is the key regulator of signal transduction of BMPs in the process of osteogenesis, and it may regulate the osteogenesis ability after transcription. Most importantly, in degenerated AF cells, overexpressed miR‐221 can attenuate the level of pSmads, which means that it can inhibit the osteogenic differentiation of degenerative AF cells and it may become a strategy for the treatment of IDD. Chen *et al*.^59^ found that the low‐expressed miR‐194‐5p can lead to an increase in CUL4A, CUL4B, and pro‐inflammatory cytokines in AF cells, and promote the occurrence of AF cell degeneration. In Hai's study^60^, the up‐regulated miR‐106a‐5p inhibited the expression of ATg7, which resulted in abnormal AF cells proliferation, autophagy, and apoptosis. But melatonin can reverse the effect of up‐regulated miR‐106a‐5p in promoting the degeneration of AF.

## Prospects and Challenges

As we all know, miRNA acts as an important gene regulator in IDD and it can be used in clinical treatment and can also be used as a biomarker for IDD diagnosis and prognosis. Based on previous research results, miRNAs have great research value and broad prospects as biomarkers or drugs for the treatment of IDD. But how to turn theoretical strategy into actual treatment? It is not difficult to find that it is feasible to delay or reverse the process of IDD by increasing, decreasing, or knocking out the expression of one or some specific miRNAs in vivo or in vitro based on animal models. However, the internal environment of the human is very complicated, and it is unclear whether such a method can take a positive effect. Therefore, it seems a feasible strategy to treat IDD by delivering the therapeutic miRNA to the local degenerated intervertebral disc through a carrier. Cheng *et al*.^5^ delivered miRNA‐21‐enriched mesenchymal stem cell exosomes to the intervertebral disc of the rat. The overexpression of miR‐21 in the intervertebral disc prevents the apoptosis process of NPCs induced by TNF‐α. Exosomal miR‐21 activates the PI3K/Akt pathway in apoptotic NPCs and inhibits the apoptosis of NPCs by inhibiting PTEN. Zhu *et al*.^61^ found that exosomes from bone marrow mesenchymal stem cells (BMSCs) may inhibit TNF‐α‐induced apoptosis and ECM degradation through the delivery of miR‐532‐5p by targeting RASSF5.

However, there are still many limitations to overcome, such as the binding of miRNA to its target gene is not completely complementary, which means that one miRNA can regulate multiple targets, at the same time, one target can be regulated by multiple miRNAs. This unconventional method of regulation reduces the specificity of miRNAs and specific diseases and will be the initial difficulty to be overcome in the clinical application of miRNAs. In addition, the development of IDD is a complicated process, so that a single aspect of research is not convincing enough, and multiple aspects will be better. Unfortunately, there are only a few miRNAs (miR‐30d, miR‐665, and miR494) that have been confirmed to participate in the IDD via the aspects mentioned above, but each of them still has multiple target genes. Therefore, a highly selective miRNA and signaling pathway that can participate in all aspects of IDD will bring huge surprises to reverse IDD. In addition, we found that the degenerated process always accompanies an inflammatory response, which is found in NPCs apoptosis, proliferation, the release of inflammatory factors, ECM degradation, and AF degeneration. We have reason to believe that this may narrow the scope of the specific miRNAs with its pathways. Furthermore, most of the previous studies have only remained at the level of pathological specimens, cells, and small animals, which are quite different from the biomechanics of the human body. Therefore, there is an urgent need for a suitable animal model similar to the human spine. Nevertheless, miRNA, as a novel gene therapy strategy for IDD, brings both hope and challenge. In short, understanding the signal pathway and mechanism of miRNA will not only help the study of the molecular mechanism of IDD but also provide new references and practical guidelines for its diagnosis and treatment.
